# Sulfur Enhances Rice Cadmium Accumulation in Organic Deficient Soil: The Significance of Incorporation with Straw

**DOI:** 10.3390/plants14223519

**Published:** 2025-11-18

**Authors:** Guoxi Wang, Lan Zhang, Yan Wang, Xia Jiang, Kun Wang

**Affiliations:** 1 Key Laboratory for Lake Pollution Control of the Ministry of Ecology and Environment, National Engineering Laboratory for Lake Pollution Control and Ecological Restoration, Chinese Research Academy of Environmental Sciences, Beijing 100012, China; wangguoxi14@mails.ucas.ac.cn (G.W.); zhanglan@craes.org.cn (L.Z.); jiangxiahp@163.com (X.J.); 2 Soil Physics and Land Management, Wageningen University & Research, 6700 AA Wageningen, The Netherlands; yanwang911021@163.com

**Keywords:** Cd accumulation, rice plant, wheat straw, sulfur, sulfate-reducing bacteria

## Abstract

Application of wheat straw could contribute to a sulfur-driven reduction in cadmium (Cd) bioavailability under reducing conditions induced by organic matter degradation. A pot experiment was conducted in organic matter deficient paddy soil under waterlogged conditions to assess the effects of sulfur (S, 30 mg kg^−1^), wheat straw (W, 1.0%), and their combination (WS) on Cd availability and accumulation in rice (*Oryza sativa* L.). Sulfur application alone increased Cd uptake in rice, whereas straw addition significantly reduced Cd accumulation, with WS achieving the greatest reduction. The mitigating effect was attributed to CdS precipitation and co-precipitation with FeS/FeS_2_ under straw amendment, as well as enhanced iron plaque formation on roots, which restricted Cd uptake. In contrast, in OM-deficient soil, sulfate promoted Cd mobilization in pore water due to limited electron supply for sulfate reduction. Compared with other sulfur forms, sulfate is more readily absorbed by rice, thereby synergistically enhancing Cd uptake by rice and promoting Cd translocation in different rice tissues. However, straw amendment supported reduction in sulfate, reducing Cd uptake by rice compared with S supplement alone. Overall, wheat straw amendment enhanced sulfur-mediated immobilization of Cd and effectively decreased Cd accumulation in rice.

## 1. Introduction

Cadmium (Cd) is recognized as a priority hazardous substance by the U.S. Department of Health and Human Services. Large quantities of Cd have been introduced into soils via mining, industrial discharges, and excessive application of fertilizers and pesticides, making Cd one of the most prevalent contaminants in Chinese paddy soils. Rice (*Oryza sativa* L.) serves as a staple food for more than 60% of China’s population. Cd is readily taken up and accumulated by rice plants, thereby posing a substantial food-chain risk to human health [1,2,3]. A recent survey of paddy soils revealed that average total soil Cd reached the concentrations of 0.45 mg kg^−1^ from 19 provinces in China, with 33.6% of the soil samples exceeding the risk screening values for agricultural land (GB15618-2018 [4]) [5]. Consequently, targeted research to interrupt the soil-to-rice transfer pathway of Cd is urgently required.

Sulfur has emerged as the fourth major macronutrient in agriculture after nitrogen, phosphorus, and potassium [6]. Sulfur can influence the fate and translocation of cadmium (Cd) in paddy soils and consequently affect Cd accumulation in rice. Several studies have reported that S supplementation can reduce Cd accumulation in rice [7,8]. One mechanism is microbial sulfate reduction under flooded, anoxic conditions: sulfate (SO_4_^2−^) is reduced by sulfate-reducing bacteria (SRB) to sulfide (S^2−^), which precipitates dissolved Cd as CdS or promotes co-precipitation with iron sulfides (FeS, FeS_2_), thereby lowering Cd availability [9,10]. In plants, S is incorporated into thiol-containing compounds such as cysteine (Cys), glutathione (GSH), and phytochelatins (PCs); PCs in particular form Cd–PC complexes sequestered in vacuoles and can limit the root-to-shoot translocation of Cd [8].

However, contrasting observations have also been reported. Excessive SO_4_^2−^ application may increase Cd accumulation in rice [7]; for example, sulfate additions can enhance Cd solubility by acidifying the rhizosphere or by forming more soluble sulfate complexes with heavy metals [10]. Water management further modulates these processes: pre-harvest drainage and the consequent oxidation of sulfide could improve Cd accumulation in the rice plant [11]. Taken together, the influence of S on Cd dynamics in paddy systems is context-dependent, governed by factors such as organic matter availability, redox conditions, microbial activity, and water regime; therefore, unilateral S application without consideration of these interacting factors may increase the risk of Cd accumulation in rice.

Soil organic matter (SOM) has been reported to modulate Cd uptake by rice, but its effect on Cd bioavailability in paddy soil remains equivocal, largely because decomposition products of SOM vary in composition and reactivity [12,13,14]. Low molecular organic acid produced during SOM decomposition can mobilize Cd and increase its bioavailability [15], whereas humic substances and other high-molecular-weight fractions tend to adsorb to mineral surfaces and complex heavy metals, thereby reducing Cd availability [16]. Moreover, sufficient SOM under flooded, anoxic conditions acts as an electron donor for microbial respiration, driving microbially mediated reduction in terminal electron acceptors (e.g., Fe^3+^, NO_3_^−^, SO_4_^2−^) [10]. This reductive sequence can promote the formation of sulfides, favoring precipitation or co-precipitation of Cd as CdS or within Fe–S phases and ultimately decreasing plant-available Cd [11].

In addition, the Cd accumulation in rice is strongly influenced by iron dynamics in the rhizosphere, notably by the formation of root iron plaque and by Fe-mediated adsorption in soils [17,18,19]. Dissolved Fe(II) in pore water is oxidized to Fe(III) by oxygen released from rice roots; the resulting Fe(III) undergoes hydrolysis and precipitates on root surfaces as iron oxides (iron plaque), which can immobilize Cd and thereby impede its uptake by the plant [7,20]. In addition, the Fe oxides, both amorphous and in microcrystalline form, possessed high specific surface and abundant sorption sites for heavy metals, increasing Cd retention in the solid phase and reducing its bioavailability to roots [21,22].

According to the Chinese soil pollution risk-control standard for agricultural land (GB15618-2018), paddy soils with Cd concentrations below the prescribed risk-screening values may still be used for crop production, provided that effective mitigation measures are implemented to ensure that harvested products comply with agricultural quality and safety standards. The present study therefore aimed to elucidate the role of sulfur and organic amendment in controlling Cd transfer from soil to rice. Specifically, the objectives were to (1) determine the effect of sulfur on the Cd availability and root iron plaque formation in the presence and absence of wheat (W) straw amendment; (2) assess how soil microbial community structure responds to combined WS application in waterlogged paddy soil; (3) quantify the influence of Cd availability and iron plaque on Cd uptake by rice plants; (4) evaluate how S affects the on Cd distribution among different rice tissues with and without straw amendment.

## 2. Materials and Methods

### 2.1. Preparation of Soil and Straw

Surface paddy soil (0–20 cm) was collected from a representative paddy field in Huaining County, Anhui Province, eastern China. The soil possessed a pH of 6.62, total S of 354.3 mg kg^−1^, Ca(H_2_PO_4_)_2_-extractable S of 15.6 mg kg^−1^, SOM of 6.5 mg g^−1^, DTPA-extractable Fe of 42.3 mg kg^−1^, total Fe of 33.2 mg kg^−1^, and total Cd of 0.17 mg kg^−1^. The soil was air-dried and passed through a 2 mm sieve, after removing roots and visible debris. A Cd-spiked soil was prepared by uniformly spraying an aqueous CdCl_2_ solution (100 mg L^−1^) and thoroughly homogenizing to achieve a target total Cd concentration of approximately 5.0 mg kg^−1^. The moistened soil was adjusted to 60% of its water-holding capacity for 14 days’ incubation to allow equilibration. The stabilized soil exhibited a pH of 6.54, total S of 341.2 mg kg^−1^, Ca(H_2_PO_4_)_2_-extractable S of 18.3 mg kg^−1^, SOM of 4.2 mg g^−1^, DTPA-extractable Fe of 46.3 mg kg^−1^, total Fe of 34.5 mg kg^−1^, DTPA-extractable Cd of 3.67 mg kg^−1^, and total Cd of 4.89 mg kg^−1^.

### 2.2. Pot Experiment and Sampling

A greenhouse pot experiment was conducted using 7.0 kg of stabilized Cd-spiked soil placed in plastic pots (240 mm Ø × 200 mm H) with an embankment height of about 12 cm. The oven-dried wheat (*Triticum aestivum* L.) straw was collected from a local farmland and ground to pass through 2 mm sieve. Four treatments were arranged (*n* = 4): control (CK), sulfate addition (S, 30 mg S kg^−1^ dry soil), straw addition (W, 1.0% *w*/*w*), and straw plus sulfate (WS). The straw was mixed into the soil and the sulfate was applied by spraying prior to filling. Basal nutrients were supplied as urea (100 mg N kg^−1^), KH_2_PO_4_ (60 mg P kg^−1^), and KH_2_PO_4_ (75.5 mg K kg^−1^). The pots stayed flooded at approximately 5 cm depth throughout the whole growth period. After revival, two seedlings were removed after establishment to standardize growth.

Rhizon soil moisture samplers (10 cm length, 0.15 μm pore size) were inserted vertically for soil pore water collection. The pore water and plant tissues (grain, leaf, stem, root) were sampled at tillering (day 14), booting (day 81), filling (day 95), and maturity (day 122); soil samples were taken at booting and filling. A portion of pore water was mixed 1:1 with sodium acetate–acetic acid buffer solution (SAOB, 2 M NaOH, 2 M C_7_H_5_O_3_Na, 0.41 M C_6_H_8_O_6_) for sulfide (S^2−^) analysis [23]. Soil was collected with a 2 cm diameter auger to 10 cm depth at a point 6 cm from the pot center. Soil subsamples were split: one fraction was stored at −80 °C for microbial community analysis, and the other was washed with deionized water to isolate fresh roots. At the booting and filling stage, one representative productive tiller was harvested per pot; portions were flash-frozen in liquid nitrogen and stored at −80 °C for organic-S analysis, and the remainder oven-dried at 70 °C for total Cd and S determinations.

### 2.3. Analytical Determinations

#### 2.3.1. Soil and Soil Pore Water

Soil pore water pH was measured directly with a pH meter (Mettler Toledo, Zurich, Switzerland), and redox potential (Eh) at 10 cm depth was recorded using an oxidation–reduction potentiometer (Dichuan, Nanjing, China). Soil organic carbon (SOC) was determined by the H_2_SO_4_–K_2_CrO_7_ oxidation method [24]. Available Cd and Fe in soil were extracted by the DTPA method following Katyal and Sharma [25]. Available soil S was extracted with 0.01 M Ca(H_2_PO_4_)_2_ and quantified by ICP–OES (iCAP PRO X, Thermo Fisher Scientific, Waltham, MA, USA) [26].

The Cd fraction of soil was performed by a sequential extraction scheme adapted from Tessier et al. [27]. Briefly, 1.0 g of air-dried soil was sequentially shaken with the following: 8 mL 1 M MgCl_2_ (pH 7) to recover the exchangeable fraction; 8 mL 1 M NaOAc (pH 5) for the carbonate-bound fraction; 20 mL 0.04 M NH_2_OH·HCl in 25% HOAc (*v*/*v*, pH 2) to extract Fe/Mn oxide-associated metals; and 20 mL 0.02 M HNO_3_ in 30% H_2_O_2_ (pH 2) to target organics-associated metals. The residual fraction was digested with freshly prepared aqua regia. Cd concentrations in the extracts were determined by ICP–OES.

Pore water sulfide (S^2−^) was measured with an ion-selective electrode using a silver/sulfide electrode (perfectION, Mettler Toledo, Zurich, Switzerland) following Balasubramanian and Pugalenthi [23]. Dissolved organic carbon (DOC) in pore water was determined with a TOC/TN analyzer (Multi N/C 2100, Analytik Jena, Jena, Germany). Dissolved Cd in pore water was quantified by inductively coupled plasma mass spectrometry (ICP–MS, iCAP RQ, Thermo Fisher Scientific), and total Fe in pore water was measured by ICP–OES. Sulfate (SO_4_^2−^) concentrations were determined by ion chromatography (Dionex ICS-2500, Thermo Fisher Scientific).

#### 2.3.2. Iron Plaque and Plant Tissues

Root iron plaque was removed using an ACA extraction solution (0.3 M sodium citrate, 10% *w/v* sodium acetate, 3 g L^−1^ ascorbic acid) and the extracted solution was analyzed for Cd and Fe by ICP–OES [28]. For total Cd and S, oven-dried plant tissues were digested with HNO_3_–H_2_O_2_ and analyzed by ICP–OES. The cysteine, glutathione, and phytochelatins (PC_2–5_) in rice tissues were determined and characterized by using HPLC (Agilent 1260 Infinity, Agilent Technologies, Santa Clara, CA, USA) with fluorescence detection [29].

#### 2.3.3. qPCR Amplification of *DsrB* Gene and 16S rRNA Sequencing

Soil DNA was extracted from 0.5 g of soil using the Power Soil DNA Isolation Kit (MoBio, Carlsbad, CA, USA) after humus removal with buffer washing. DNA quality was checked on 1% agarose gels, and its concentration and purity were determined with a Nano Drop 2000 (Thermo Fisher Scientific). The sulfite reductase gene (*DsrB*) was PCR-amplified using primers DSRp2060F (CAACATCGTYCAYACCCAGGG) and DSR4R (GTGTAGCAGTTACCGCA) under the following conditions: 94 °C for 4 min; 35 cycles of 94 °C for 1 min; 55 °C for 1 min; 72 °C for 1 min; and 72 °C for 10 min. Amplicons were visualized on 1.5% ethidium bromide-stained agarose gels. Standard curves were generated from five replicates of 10-fold plasmid dilutions containing cloned targets.

The V3–V4 hypervariable regions of the 16S rRNA were PCR-amplified using the following primers: 338F (5′-ACTCCTACGGGAGGCAGCAG-3′) and 806R (5′-GGACTACHVGGGTWTCTAAT-3′) [30]. The amplification was carried out with an initial denaturation of 95 °C for 3 min, followed by 25 cycles with 95 °C for 30 s, 48 °C for 30 s, 72 °C for 45 s, and a final extension at 72 °C for 10 min. Amplicons were separated on a 2% agarose gel and excised for further analysis. High-throughput sequencing was performed on an Illumina MiSeq 2500 platform (Majorbio, Shanghai, China) assigned to a bacterial database of silva 128. The 16S rRNA sequencing data were analyzed using QIIME 1.7.0 software. Operational taxonomic units (OTUs) were classified at the 97% sequence similarity threshold and a representative sequence for each OTU was aligned using the Ribosomal Database Project classifier (version 2.2) and Green Gene database.

### 2.4. Statistical Analysis

The pe + pH value of the soil pore water was calculated using the formula Eh(V)/0.059 + pH (at 25 °C) [31]. Differences in soil pore water characteristics and rice plant tissues among treatments were assessed using one-way analysis of variance (ANOVA) followed by Tukey’s test at a significance level of *p* < 0.05. Correlation analyses were performed using Pearson’s test at *p* < 0.05 (SPSS 23, IBM, Armonk, NY, USA).

## 3. Results

### 3.1. Soil Pore Water Properties and Cd Fractions

**Figure 1:**
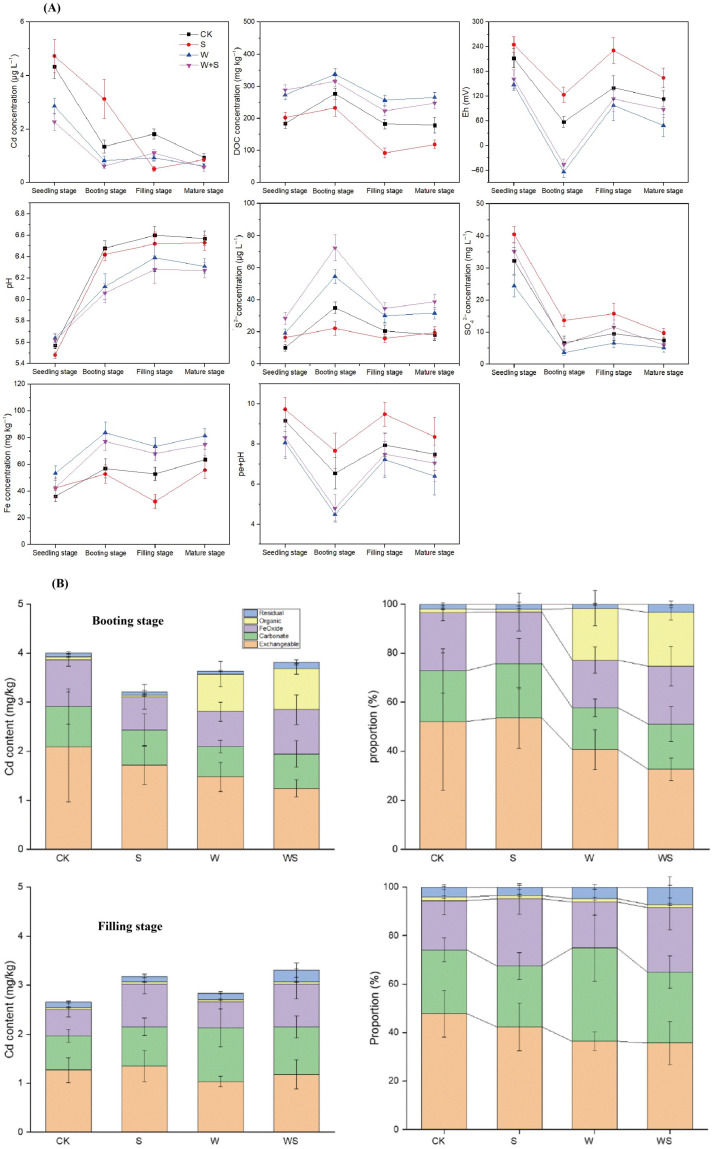
Physicochemical characteristics of pore water and Cd speciation in Cd-contaminated paddy soil amended by sulfur (S), wheat (W), and their incorporations (WS) compared to without amendment control (CK) at different rice growth stages. (**A**) Dynamic of Eh, pH, DOC, pe + pH, S^2−^, SO_4_^2−^, and Fe in soil pore water; (**B**) distribution of Cd speciation in the paddy soil.

The pH of soil pore water increased across all treatments from the seedling to the filling stage, after which it stabilized and remained constant until rice maturity (Figure 1A). From the booting stage onward, the pH values of W-amended soils were significantly lower than those of unamended soils regardless of S addition (Figure 1A). The variations in soil Eh and pe + pH followed a similar trend, decreasing from seedling to the booting stage and subsequently increasing from booting to the filling stage (Figure 1A). Throughout the entire rice growth period, the Eh and pe + pH values in W-amended soils were consistently and significantly lower than those in unamended soils (Figure 1A). Moreover, S application significantly increased both Eh and pe + pH values in the soils without W amendment (Figure 1A). Pore water SO_4_^2−^ declined from tillering to the booting stage, followed with a pronounced rebound at the filling stage; in contrast, the S^2−^ concentration of soil pore water increased from seedling to the booting stage, and subsequently decreased from booting to the filling stage (Figure 1A). SO_4_^2−^ was observed in higher concentrations in both W- or without W-amended soil under S addition (Figure 1A). W amendment significantly increased the S^2−^ concentration of soil pore water irrespective of S addition, and, in particular, the S^2−^ concentration of soil pore water remained the highest among all treatments throughout the entire rice growth period (Figure 1A).

The concentration of dissolved Cd for all treatments was observed in the remarkable decline from seeding to the booting stage, but thereafter showed divergent trends, decreasing in S treatment, but increasing in CK, W, and WS treatments from booting to the filling stage (Figure 1A). At the booting stage, the S treatment possessed the highest average Cd concentration of 3.26 ± 0.68 μg·L^−1^, while in the WS treatment was observed the lowest average Cd concentration of 0.72 ± 0.07 μg·L^−1^ (Figure 1A). However, the highest average Cd concentration of 1.86 ± 0.14 μg·L^−1^ was observed in W treatment, whereas it was lowest in S treatment with an average concentration of 0.63 ± 0.08 μg·L^−1^ at the filling stage (Figure 1A). In terms of soil Cd, the exchangeable, carbonate-bound, and Fe/Mn oxide-bound Cd were the dominant fractions for all treatments at both booting and filling stages (Figure 1B). At the booting stage, W amendment decreased both in proportion and concentration of exchangeable and carbonate Cd, but increased those of organic Cd in the soil irrespective of S addition; S addition increased the proportion and concentration of Fe/Mn oxide Cd under W amendment, but decreased those of Fe/Mn oxide Cd without W amendment (Figure 1B). At the filling stage, S addition increased the concentration of exchangeable and Fe/Mn oxide Cd in both with and without W-amended soil. Furthermore, combined S and W application enhanced residual Cd at both booting and filling stages, and W amendment increased the carbonate Cd concentration in the soil irrespective of S addition (Figure 1B). Whether at the booting or filling stage, combined application of W and S showed the highest concentration and proportion of residual Cd among all treatments (Figure 1B).

### 3.2. Soil SRB and DsrB Gene Abundance

The relative abundance of soil SRB and the abundance of the *DsrB* gene were assessed at the booting stage when the pe + pH underwent a turning point in the waterlogged paddy soil. W amendment significantly increased the abundance of *DsrB* gene irrespective of S addition, and addition of S further increased the abundance of *DsrB* gene in W-amended soil. In the WS treatment was observed the highest average *DsrB* gene abundance of 12.65 ± 2.21 × 10^7^ copies·g^−1^ among all treatments (Table 1). Moreover, W amendment increased the relative abundance of *Geobacter*, *Desulfovibrio*, *Desulfobulbus*, and total SRB irrespective of S addition, and addition of S further increased the relative abundance of those bacteria in W-amended soil. The highest average relative abundance of *Geobacter*, *Desulfovibrio*, *Desulfobulbus*, and total SRB were also observed in WS treatment, with the values of 2.69 ± 0.29 × 10^7^, 0.87 ± 0.35 × 10^7^, 0.22 ± 0.15 × 10^7^, and 4.38 ± 0.74 × 10^7^ copies·g^−1^, respectively (Table 1).

### 3.3. Cd and Fe in Iron Plaque of Root Surface

**Figure 2:**
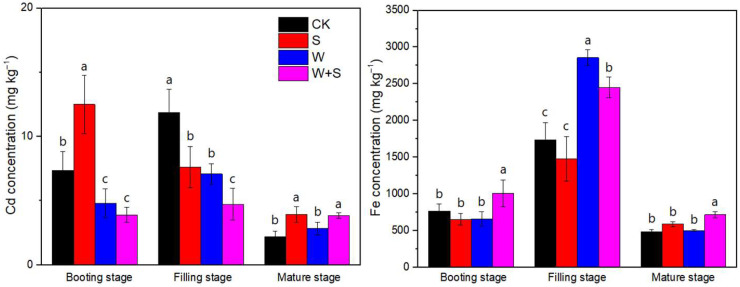
ACA-extractable Fe and Cd (mg·kg^−1^ DW) in iron plaque of rice root under sulfur (S), wheat straw (W), and their incorporations (WS) amendment compared to without amendment control (CK) at the booting, filling, and mature stages; ACA, acetic acid. Different letters represent significant differences between treatments at *p* < 0.05.

The Cd concentration of iron plaque decreased from booting to the mature stage in S treatment, while in CK, W, and WS treatments, there was observed an increase from booting to the filling stage, followed with a decrease from filling to the mature stage (Figure 2). At the booting stage, W amendment significantly reduced the Cd concentration of iron plaque irrespective of S addition; the highest average iron plaque concentration was 12.6 ± 2.4 mg·kg^−1^ dry root (Figure 2). At the filling stage, both S addition and W amendment reduced the Cd concentration of iron plaque compared with CK, and the S addition further decreased the concentration of iron plaque Cd in W-amended soil; in WS was observed the lowest iron plaque Cd concentration of 4.9 ± 1.1 mg·kg^−1^ dry root among all treatments (Figure 2). The Fe concentration of iron plaque increased from booting to the filling stage, and subsequently decreased from filling to the mature stage for all treatments (Figure 2). At the filling stage, W amendment significantly enhanced the iron plaque Fe concentration irrespective of S addition, while S addition reduced the Fe concentration in W-amended soil; the highest iron plaque Fe concentration of 2847 ± 136 mg·kg^−1^ dry root was observed in W treatment (Figure 2).

### 3.4. Total S and Organic S Compounds in Rice Plant Tissues

S concentration in rice tissues varied strongly with growth stage and treatments (Table 2). At the booting stage, leaves showed the highest S concentration of 2553.2 ± 78.9 and 2487.5 ± 124.9 mg·kg^−1^ in WS and S treatments, respectively, both significantly higher than CK and W (*p* < 0.05) (Table 2). The highest S concentration of the root of 731.9 ± 46.3 mg·kg^−1^ was observed in S treatment and the lowest concentration of 648.6 ± 48.8 mg·kg^−1^ in CK (Table 2). During the filling stage, S treatment markedly increased S concentration in the grain (996.6 ± 88.6 mg·kg^−1^), leaf (2095.1 ± 93.5 mg·kg^−1^), and stem (1243.4 ± 133.6 mg·kg^−1^) compared with other treatments, while W produced the lowest S concentration in the stem and grain; there were no significant differences among all treatments for root S concentration (Table 2). At maturity, brown rice S showed the highest concentration of 936.1 ± 68.9 mg·kg^−1^ compared with other treatments, and husk S concentration was elevated in WS (587.3 ± 76.2 mg·kg^−1^) and S (526.1 ± 62.7 mg·kg^−1^) treatments compared to CK and W (Table 2). The S concentration in the leaf (1605.2 ± 197.7 mg·kg^−1^) and stem (848.2 ± 37.6 mg·kg^−1^) at maturity were significantly greater in S treatment than in other treatments (Table 2). Root S concentration at maturity was highest in W and S treatments, while the lowest concentration was shown in WS (857.9 ± 27.8 mg·kg^−1^) treatment (Table 2).

**Figure 3:**
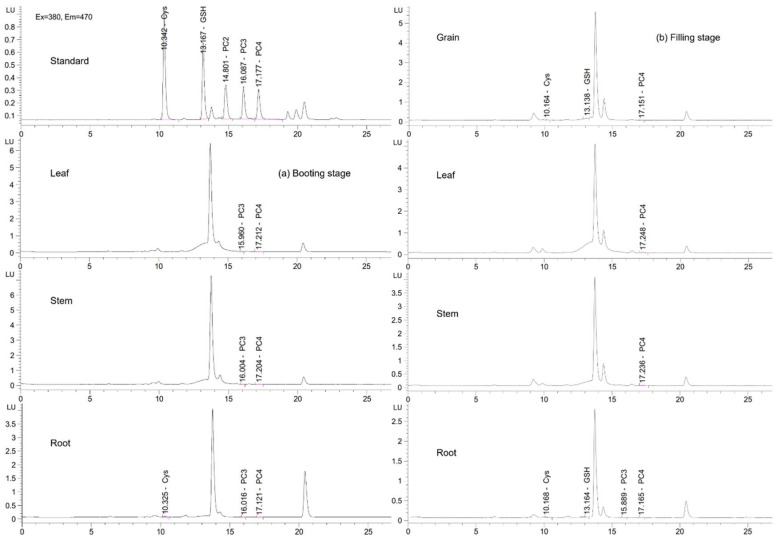
Different forms of organic S compounds (cysteine (Cys), glutathione (GSH), and phytochelatins (PCs)) determined by high-performance liquid chromatography (HPLC) in rice root, stem, leaf, and grain at the booting (**a**) and filling (**b**) stage, respectively.

Concentrations of key organic S compounds, cysteine (Cys), glutathione (GSH), and phytochelatins (PC_2_, PC_3_, PC_4_), were assessed in rice tissues at both booting and filling stages (Figure 3). The concentrations of Cys, GSH, and PCs in rice tissues varied markedly between treatments and growth stages (Table 3). At the booting stage, no grain was present, and the Cys and GSH concentrations in leaves and stems were below the detection limit, while PCs were detectable in all tissues. The concentration of root PCs ranged from 51.1 to 59.2 mg·kg^−1^ (Table 3). During the filling stage, the grain Cys concentration had a range of 62.3 to 67.6 mg·kg^−1^, and there were no significant differences across treatments; the W treatment showed the highest grain GSH concentration of 95.3 ± 8.3 mg·kg^−1^ and the S treatment showed the highest grain PCs concentration of 92.42 ± 4.3 mg·kg^−1^ (Table 3). Leaf PCs concentrations were substantially enhanced by S addition (165.1 ± 31.5 mg·kg^−1^) compared with CK (81.74 ± 18.2 mg·kg^−1^) and other treatments, whereas stem PCs showed no significant differences among treatments with the concentration ranging from 77.5 to 86.4 mg·kg^−1^ (Table 3). In the root, Cys and GSH showed the highest concentrations of 78.1 ± 7.2 and 76.5 ± 3.3 mg·kg^−1^ in S treatment, and the PCs showed no significant differences among treatments with a concentration range of 135.0 to 147.4 mg·kg^−1^, indicating consistent detoxification activity in the root (Table 3).

### 3.5. Cd Concentration in Rice Plant Tissues

**Figure 4:**
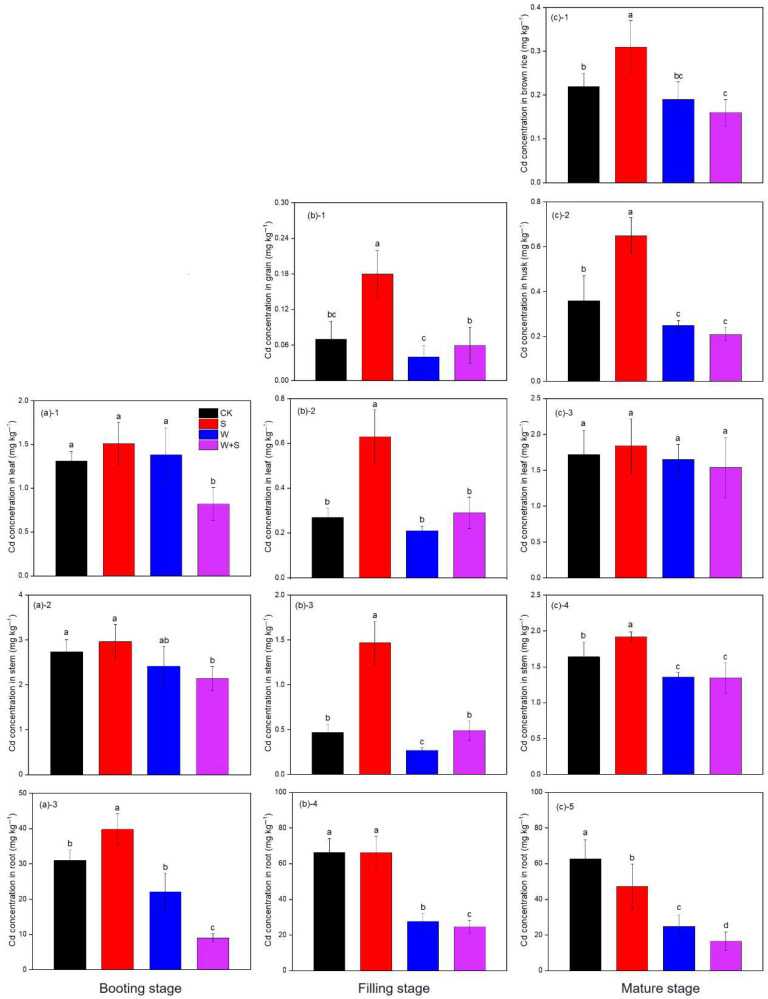
Cd concentration in different rice plant tissues under sulfur (S), wheat straw (W), and their incorporations (WS) amendment compared to without amendment control (CK) at the booting (**a**), filling (**b**), and mature (**c**) stages. Different letters represent significant differences between treatments at *p* < 0.05. (a)-1, (a)-2 and (a)-3, represent Cd concentration in leaf, stem and root respectively; (b)-1, (b)-2, (b)-3 and (b)-4, represent Cd concentration in grain, leaf, stem and root respectively; (c)-1, (c)-2, (c)-3, (c)-4 and (c)-5, represent Cd concentration in husk, brown rice, leaf, stem and root respectively.

At the mature stage, the Cd concentration in brown rice ranged from 0.16 mg·kg^−1^ to 0.31 mg·kg^−1^ (Figure 4). The S application alone increased Cd concentration in the brown rice compared to CK. In addition, the brown rice showed lower Cd concentration in W-amended treatments irrespective of S addition, and the lowest Cd concentration of brown rice was observed in the WS treatment (Figure 4). At the filling stage, S application alone significantly increased the Cd concentration of the grain, leaf, and stem in comparison with the CK, and showed the highest concentration among all treatments, while root Cd concentration was observed to be lower in W-amended soil irrespective of S addition (Figure 4). At the booting stage, S addition reduced the leaf Cd concentration in W-amended soil, and the lowest leaf Cd concentration was shown in WS treatment; W amendment reduced the root Cd concentration irrespective of S addition, but the S addition in W-amended soil further reduced the Cd concentration compared with W treatment (Figure 4).

### 3.6. Correlation Between Environmental Factors and Microbial Communities

**Figure 5:**
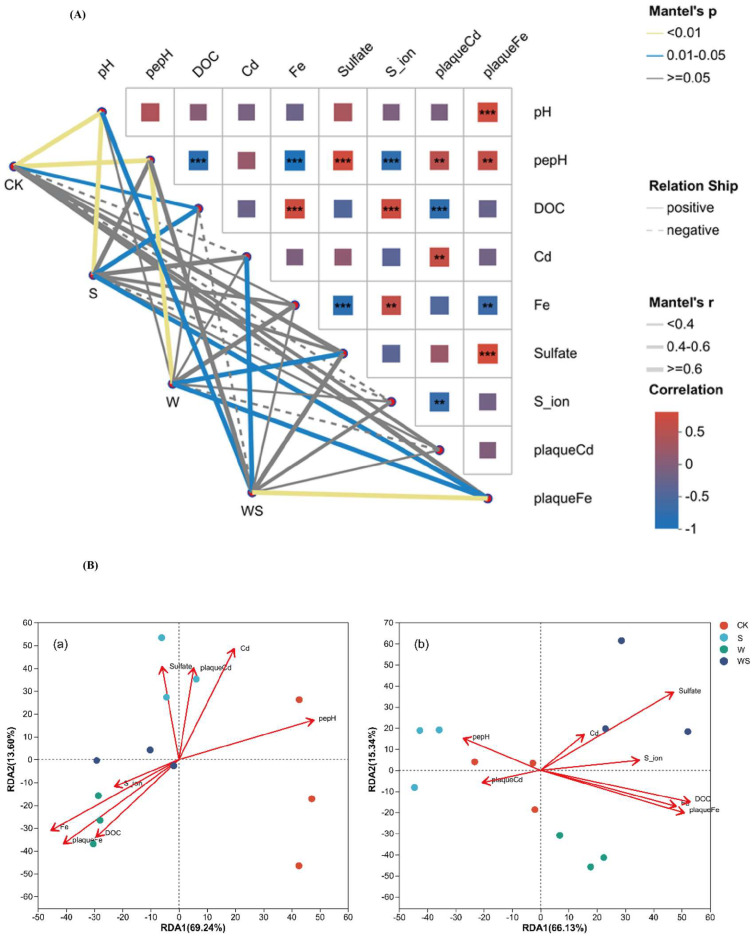
(**A**) The mantel test heatmap for correlation between environmental factors and microbial communities during the rice growth, ** and *** represent significance of *p* ≤ 0.01 and *p* ≤ 0.001, respectively. (**B**) Redundancy analysis (RDA) for relationship between environmental factors and soil microbial communities at booting (**a**) and filling (**b**) stages, respectively. CK, control group; S, individual sulfate application; W, individual wheat straw application; WS, combined application of W and S.

Mantel tests and redundancy analysis (RDA) were performed to examine the relationships between environmental factors and microbial community structures (Figure 5). Mantel analysis revealed that SO_4_^2−^, Cd, plaque Cd, and DOC were strongly and positively correlated with microbial community shifts (r ≥ 0.6, *p* < 0.05). SO_4_^2−^ and Cd showed highly significant associations (*p* < 0.01), suggesting that these factors play a key role in shaping microbial structure (Figure 5A). The RDA axes explained a large proportion of the variation at the booting (a: RDA1 = 69.24%, RDA2 = 18.96%) and filling (b: RDA1 = 66.13%, RDA2 = 13.94%) stages, respectively (Figure 5B). At the booting stage, CK samples were clustered separately while W and WS treatments clustered closely; at the filling stage, S and WS treatments were clustered distinctly. The result suggested that W amendment exerted a stronger influence on community composition than S application alone (Figure 5B). The RDA further indicated that pe + pH, S^2−^, Fe, plaque Fe, and DOC were the primary drivers of microbial community at both booting and filling stages, and the SO_4_^2−^ and plaque Cd affect the microbial community at the filling stage as well (Figure 5B).

### 3.7. Co-Occurrence Network Between Microbial Genera and Environmental Factors

**Figure 6:**
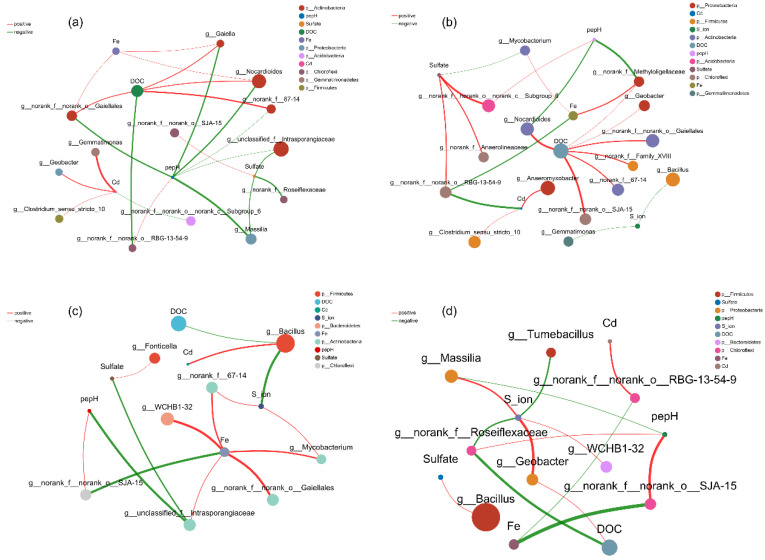
The collinearity network analysis for the correlation between microbial species (phyla level) and environmental factors in the paddy soil amended by sulfur (**a**), wheat (**b**), and their incorporations (**c**) compared to without amendment control (**d**). The size of nodes represents the abundance of species; the thickness of the edges indicates the magnitude of correlation coefficients.

Co-occurrence networks were constructed to assess microbial–environment interactions under different soil amendments (Figure 6). In S treatment (Figure 6a), strong positive correlations were observed between SO_4_^2−^ and genera such as *Nocardioides* and *Geobacter*, indicating the enrichment of sulfur-cycling and metal-reducing bacteria. DOC, Fe, and Cd positively correlated with *Gaiella* and *Intrasporangiaceae*, while negative associations with unclassified taxa suggested selective pressure from heavy metals. In W treatment (Figure 6b), DOC and pe + pH positively correlated with *Bacillus*, *Mycobacterium*, and *Anaeromyxobacter*, reflecting enhanced organic matter degradation and redox activity; negative correlations, particularly between SO_4_^2−^ and *Geobacter*, indicated shifts in community structure due to organic inputs. WS treatment (Figure 6c) generated distinctive patterns, with *Bacillus* linked to DOC, Fe, and Cd, and *Fonticella* associated with S and pe + pH, suggesting adaptive responses to both organic and inorganic amendments. Several negative correlations with rare taxa implied competitive exclusion or niche differentiation. CK (Figure 6d) exhibited a simpler, less connected network, with dominant positive correlations involving *Geobacter*, *Massilia*, and *Bacillus*, but lower overall connectivity, indicating weaker environmental modulation of microbial communities.

### 3.8. Correlation of Cd and Sulfur Accumulation in Rice Tissues with Soil and Plaque Properties

**Figure 7:**
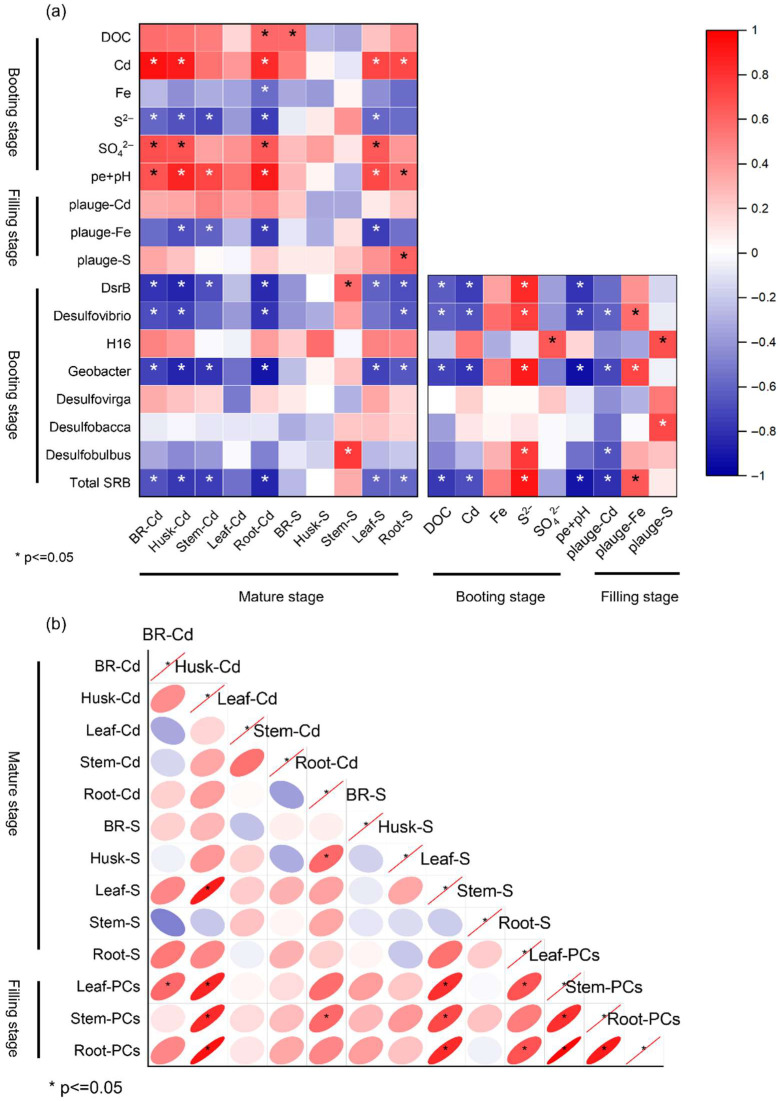
Correlation between concentration of Cd and S in different rice plant tissues and properties of iron plaque, as well as the properties of soil pore water (**a**), and the correlation among the concentration of Cd, total S, and PCs in different rice plant tissues (**b**). * represents significant correlations at *p* < 0.05.

Correlation analysis across growth stages revealed relationships between Cd and S dynamics in rice tissues and their associations with iron plaque and soil pore water chemistry (Figure 7). At maturity, Cd in brown rice (BR-Cd), husk (Husk-Cd), and roots (Root-Cd) positively correlated with DOC, Fe, and SO_4_^2−^, but negatively with plaque Fe and several SRB genera (*Desulfovibrio*, *Desulfobulbus*, *Desulfobacteraceae*), whereas S in rice tissues correlated positively with soil pore water S^2−^ and negatively with pe + pH (*p* < 0.05) (Figure 7a). At the booting and filling stages, root and plaque properties were more tightly linked to microbial activity, with *Geobacter* and *Desulfobacca* showing significant correlations with Fe and Cd, and plaque-bound Cd and Fe negatively correlated with SRB abundance, supporting SRB-mediated reduction in Cd bioavailability (Figure 7a). Interrelationships between Cd, S, and phytochelatins (PCs) indicated strong positive correlations between S and PCs in roots, stems, and leaves at the filling stage, particularly in roots (*p* < 0.05), suggesting S-enhanced PCs synthesis for Cd detoxification (Figure 7b). Cd and S concentrations were also significantly correlated in multiple tissues, notably BR-Cd with BR-S and Husk-Cd with Husk-S, implying coupled uptake or detoxification under Cd stress (Figure 7b).

## 4. Discussion

### 4.1. Effect of Sulfur on the Cd Availability Under Straw Amendment During Rice Growth

Cadmium solubility in paddy pore water was strongly controlled by redox conditions in this study (Figure 1). Cd declined from tillering to booting as Eh decreased, consistent with classic observations that reducing conditions favor the formation of S- and Fe-bound Cd phases and therefore lower dissolved Cd [9]. Fulda et al. demonstrated quantitatively that progressive reduction drives rapid decreases in soluble Cd as sulfide phases form, and X-ray spectroscopic work supports a shift from labile Cd to S-coordinated and residual species during reduction [9,32]. Wheat straw (W) amendment markedly enhanced this immobilization cascade in our pots: W alone lowered pore water Cd, and combined W + S (WS) produced the largest suppression during the tillering–booting interval (Figure 1). This outcome is in line with recent studies that report the synergistic effects of organic amendments plus sulfate in decreasing Cd mobility and grain uptake via the provision of electron donors that stimulate sulfate-reducing bacteria (SRB), promotion of S^2−^ formation and biogenic CdS/FeS precipitation, and increased sorption to newly formed organo-mineral phases [31,32,33,34,35].

Multiple recent studies emphasize the central role of dissolved organic matter (DOM/DOC) in setting the direction and magnitude of these responses. High DOC and labile OM accelerate microbial respiration and drive the sequential reduction of O_2_, Fe^3+^, NO_3_^−^, and SO_4_^2−^, thereby favoring sulfide production and Cd immobilization; conversely, low SOM limits electron supply, suppresses SRB activity, and can leave added SO_4_^2−^ unreduced and available to form soluble complexes with Cd [1,36]. Recent studies confirm that DOM amendments modulate Cd mobility in waterlogged soils largely through these biogeochemical pathways [34,37,38].

The divergent behavior observed under S application alone, where SO_4_^2−^ increased pore water Cd at the booting stage, accords with the conditional responses reported in recent studies (Figure 1). Several studies show that the efficacy of sulfate amendments depends critically on SOM content and microbial capacity for sulfate reduction: where labile OM is sufficient, sulfate addition promotes sulfide precipitation and reduces Cd; where SOM is limited, sulfate may increase Cd solubility and plant uptake. Thus, our finding that S alone raised pore water Cd in SOM-deficient soil is consistent with an emerging consensus that sulfur effects are context-dependent [34,35,37]. Mechanistically, the microbial mediation of these processes is well supported: recent studies show that SRB (and associated *DsrB* gene abundance) are tightly linked to S^2−^ production and to decreases in dissolved Cd, and that co-amendment with labile carbon (e.g., straw) amplifies SRB responses and Cd immobilization [34,39]. Network and spectroscopic evidence also indicate that re-oxidation (e.g., during rising Eh or pre-harvest drainage) can remobilize Cd from sulfide pools, highlighting the potential transient nature of sulfide-bound protection [10,38].

The findings of previous studies are consistent with our results in three key aspects: (1) redox decline under flooding favors S- and Fe-mediated Cd immobilization [9]; (2) co-application of labile organic carbon (wheat straw) with sulfate most reliably promotes SRB activity, sulfide formation, and Cd sequestration [34,38,40]; and (3) in SOM-deficient soils, sulfate alone can increase Cd mobility and plant uptake because of the insufficient electron donors to drive sulfate reduction.

### 4.2. Effect of Sulfur on Iron Plaque of Rice Root Under Straw Amendment

Iron plaque, composed of crystalline and amorphous Fe oxides, showed maximal Fe content at the filling stage across treatments, consistent with previous reports [20,22]. Straw-amended soils exhibited higher plaque Fe, correlated with elevated Fe concentrations in pore water, suggesting enhanced Fe^2+^ mobilization and subsequent oxidation to Fe^3+^ on the root surface [41,42]. Sulfate application, however, reduced plaque Fe, possibly due to the coordination of SO_4_^2−^ with Fe^2+^, limiting oxidation [43].

Plaque Cd concentrations peaked at filling for all treatments, reflecting Cd transfer from soil to the root surface during maximal nutrient uptake. W amendment consistently lowered plaque Cd at booting, likely due to reduced pore water Cd and lower pe + pH, whereas S application increased plaque Cd at booting, reflecting higher pore water Cd. By the filling stage, higher plaque Fe in W-amended soils facilitated greater Cd adsorption, whereas S application decreased plaque Cd, likely due to the SO_4_^2−^-induced root uptake of plaque-bound Cd. These results align with previous findings that iron plaque serves as both a sink and source of Cd for rice roots, modulating Cd translocation to aboveground tissues [6]. Notably, root Cd content was highest under S treatment at filling, supporting the role of sulfate in enhancing Cd uptake under low SOM conditions.

### 4.3. Availability of Soil Cd Induced by Microbial Community and SRB Under Combined Application of Sulfur and Straw

Soil microbes play a pivotal role in regulating Cd bioavailability through adsorption, mineralization, and precipitation processes [20]. In the present study, microbial community composition shifted with rice growth stages, reflecting changes in the rhizosphere environment during the booting–filling stages. During this reproductive phase, rice roots preferentially acquired available N, P, and S via rhizospheric oxygenation and organic matter mineralization, which corresponded with increased Eh and pe + pH and decreased DOC in pore water (Figure 1A).

At the booting stage, W-amended soils exhibited microbial communities positively associated with S^2−^, DOC, pore water Fe, and root iron plaque Cd, but negatively correlated with pore water Cd (Figure 5B). This pattern likely reflects the strong reducing conditions generated by the rapid decomposition of organic matter during early growth, which promotes Fe^2+^ mobilization and SRB-mediated reduction of SO_4_^2−^ to S^2−^ [42]. By the filling stage, microbial communities in WS-treated soils showed positive associations with SO_4_^2−^ and pore water Cd, suggesting that microbial-driven oxidation of CdS and associated sulfide compounds released Cd and sulfate into the pore water. Overall, WS treatment maintained a positive correlation between microbial communities and pore water Cd throughout growth, indicating that combined straw and sulfur application enhanced the microbial modulation of Cd dynamics (Figure 5A).

Network analysis at the genus level revealed a positive correlation between *Geobacter* and S^2−^ in WS soils, highlighting the central role of sulfate-reducing bacteria (SRB) in converting SO_4_^2−^ to S^2−^ and mediating Cd immobilization (Figure 6). Indeed, pore water Cd was lowest in WS treatments at the booting stage (Figure 1), consistent with sulfide precipitation mechanisms. SRB, obligate anaerobes that perform dissimilatory sulfate reduction, use sulfate as a terminal electron acceptor to generate sulfide, facilitating CdS precipitation and co-precipitation with FeS/FeS_2_ [44,45,46].

Straw application significantly increased the relative abundance of SRB genera, while sole sulfur supply decreased it. The combined application of straw and sulfur resulted in the highest SRB abundance, with genera such as *Geobacter*, *Desulfovibrio*, *H16*, *Desulfobacca*, and *Desulfovirga* substantially enriched compared to CK (Table 1). Correspondingly, *DsrB* gene abundance, a key functional marker for dissimilatory sulfate reduction, was markedly higher under combined amendments. Correlation analysis further indicated that pore water Cd positively correlated with S^2−^ but negatively with *DsrB* gene abundance and SRB relative abundance (Figure 7), supporting the conclusion that SRB activity is a primary driver reducing Cd availability in paddy soil during early rice growth.

### 4.4. Effect of Sulfate on Cd Accumulation in Rice Under Straw Amendment

This study showed that W application reduced Cd accumulation in brown rice relative to CK regardless of S addition; notably, only the WS treatment lowered brown rice Cd to below 0.2 mg·kg^−1^ (the maximum permissible limit in GB2762-2012 [47]). At the booting stage, Cd concentrations in brown rice and roots were positively correlated with soil pore water Cd, whereas at the filling stage brown rice Cd was negatively correlated with Fe in root iron plaque (Figure 7a). These results indicate that a reduction in soil-available Cd together with enhanced iron plaque formation under the WS treatment were key factors limiting Cd transfer into edible grain. This conclusion agrees with recent pot- and field-based studies reporting the synergistic effects of organic amendments (e.g., straw) and sulfur in decreasing soil Cd bioavailability and promoting iron plaque formation [34,38].

We observed a significant negative correlation between brown rice Cd and both the relative abundance of sulfate-reducing bacteria (SRB) and *DsrB* gene abundance in soil (Figure 7a). This pattern suggests that microbially mediated sulfate reduction and the subsequent precipitation of metal sulfides were principal mechanisms lowering Cd bioavailability during early growth. Previous studies have shown that SRB activity can lower pore water redox potential (Eh) and promote sulfide production, thereby immobilizing Cd as insoluble sulfides [48,49]. Moreover, the presence of organic matter (such as straw) provides labile electron donors that can enhance SRB activity and thereby amplify sulfur-driven Cd immobilization [32,38].

In contrast to multiple earlier reports that sulfur application decreases plant Cd accumulation via enhanced iron plaque formation and thiol-mediated sequestration, sole S application in our experiment increased brown rice Cd (Figure 4c). In soils deficient in organic matter or other available electron donors, applied SO_4_^2−^ may not be effectively reduced to S^2−^; instead, SO_4_^2−^ can persist in the pore water or act as a ligand that promotes desorption of Cd from solid phases into the soil solution, thereby increasing pore water Cd. Some studies have reported short-term increases in Cd availability following sulfate addition under SOM-poor conditions [50,51]. Under such conditions, lack of coupling between sulfate addition and microbial reduction prevents CdS formation and may even enhance soluble Cd pools.

Moreover, S addition alters plant sulfur metabolism and physiology, which can change internal Cd redistribution. Sulfur is required for the synthesis of cysteine (Cys), glutathione (GSH), and phytochelatins (PCs); these thiol-containing molecules coordinate Cd via sulfhydryl (–SH) groups and commonly promote vacuolar sequestration in roots, thereby limiting root-to-shoot translocation [52]. However, under certain circumstances—such as increased pools of low-molecular-weight thiols, changes in subcellular compartmentalization or transporter activity, or enhanced transpiration/xylem flow—Cd–thiol complexes may become more mobile and facilitate Cd transfer to aerial tissues. Transporters implicated in root-to-shoot Cd movement (e.g., OsZIP2 and other transporters) and long-distance xylem/phloem dynamics can also influence this process. In our study, sole S treatment produced the highest pore water Cd at booting and significantly elevated root Cys and GSH concentrations at the filling stage (Table 3), consistent with a scenario in which increased soluble soil Cd together with altered plant sulfur metabolism enhanced upward Cd translocation [52,53].

Generally, when sulfur addition is coupled with organic matter amendment, sulfur tends to reduce Cd bioavailability and grain accumulation via sulfide precipitation, promotion of iron plaque formation, and enhanced vacuolar sequestration [38,49]. Conversely, in SOM-poor soils or where active sulfate reduction is absent, sole sulfate application may transiently increase pore water Cd and, by modifying plant sulfur metabolism and transport processes, promote Cd translocation to aboveground tissues [48,51,54].

## 5. Conclusions

In this study, rice was cultivated in Cd-contaminated paddy soil under waterlogged conditions. Wheat straw (W) amendment reduced Cd accumulation in brown rice regardless of S addition, while combined W and S application further decreased Cd accumulation compared to W alone. In W-amended soil, S application promoted the precipitation of CdS and/or co-precipitation of Cd with FeS/FeS_2_, driven by the microbial reduction of SO_4_^2−^ to S^2−^ under waterlogging. In contrast, sole S application in non-W-amended soil significantly increased Cd accumulation in brown rice. The lack of soil organic matter (SOM) limited sulfate reduction, resulting in higher Cd concentrations in soil pore water and enhanced co-uptake of sulfate and Cd by rice roots. These findings suggest that the effect of S on Cd accumulation in rice depends on SOM content, indicating that individual S application should be avoided in Cd-contaminated, SOM-deficient paddy soils. Overall, this study provides insights into safe rice cultivation in Cd-contaminated paddy soils.

## Figures and Tables

**Table 1:** Relative abundance (%) of dominant SRB genera and abundance of *DsrB* gene (10^7^ copies·g^−1^) in paddy soil at the booting stage.

	CK	S	W	WS
*DsrB* gene	5.64 ± 0.89 c	3.32 ± 1.17 d	7.93 ± 1.46 b	12.65 ± 2.21 a
*Geobacter*	1.47 ± 0.08 b	1.07 ± 0.10 c	2.33 ± 0.31a	2.69 ± 0.29 a
*Desulfovibrio*	0.42 ± 0.06 b	0.25 ± 0.11 c	0.70 ± 0.24 a	0.87 ± 0.35 a
*H16 ^‡^*	0.07 ± 0.01 c	0.31 ± 0.00 a	0.13 ± 0.04 b	0.20 ± 0.09 b
*Desulfovirga*	0.13 ± 0.08 a	0.10 ± 0.05 a	0.15 ± 0.07 a	0.17 ± 0.04 a
*Desulfobacca*	0.05 ± 0.01 b	0.09 ± 0.04 a	0.08 ± 0.04 a	0.09 ± 0.05 a
*Desulfobulbus*	0.01 ± 0.01 c	0.06 ± 0.02 b	0.08 ± 0.04 b	0.22 ± 0.15 a
Total SRB	2.25 ± 0.14 c	2.05 ± 0.29 c	3.60 ± 0.39 b	4.38 ± 0.74 a

CK, without addition; W, addition of wheat straw; S, addition of sulfate; WS, combined application of wheat straw and sulfate; different letters represent significant differences between treatments at *p* < 0.05. *^‡^*, a genus of SRB, belong to f_*Desulfurellaceae*, o_*Desulfurellales*.

**Table 2:** Total S concentration (mg·kg^−1^) of rice tissues at the booting, filling, and mature stages.

	CK	S	W	WS
Booting stage				
Leaf	2286.1 ± 137.1 b	2487.5 ± 124.9 a	2270.2 ± 164.5 b	2553.2 ± 78.9 a
Stem	1537.2 ± 88.5 a	1500.5 ± 151.2 a	1581.4 ± 83.2 a	1550.3 ± 102.1 a
Root	648.6 ± 48.8 b	731.9 ± 46.3 a	685.2 ± 64.8 a	670.2 ± 42.2 ab
Filling stage				
Grain	684.32 ± 40.9 c	996.6 ± 88.6 a	584.4 ± 58.8 d	792.2 ± 46.3 b
Leaf	1058.3 ± 82.9 bc	2095.1 ± 93.5 a	935.3 ± 61.9 c	1183.3 ± 103.1 b
Stem	823.13 ± 51.7 b	1243.4 ± 133.6 a	615.6 ± 38.6 c	759.7 ± 66.1 b
Root	933.69 ± 35.2 a	1004.2 ± 86.1 a	927.6 ± 54.8 a	960.9 ± 81.4 a
Mature stage				
Brown rice	851.1 ± 72.4 b	936.1 ± 68.9 a	824.2 ± 72.4 b	832.5 ± 46.3 b
Husk	471.1 ± 46.3 b	526.1 ± 62.7 a	412.7 ± 45.4 b	587.3 ± 76.2 a
Leaf	1201.8 ± 200.5 b	1605.2 ± 197.7 a	1019.3 ± 173.7 bc	1062.8 ± 78.6 bc
Stem	781.1 ± 22.9 b	848.2 ± 37.6 a	745.9 ± 42.5 b	726.3 ± 35.7 b
Root	863.5 ± 45.2 ab	925.6 ± 40.3 a	959.9 ± 78.6 a	857.9 ± 27.8 b

CK, without addition; W, addition of wheat straw; S, addition of sulfate; WS, combined application of wheat straw and sulfate; different letters represent significant differences between treatments at *p* < 0.05.

**Table 3:** The concentration (mg·kg^−1^) of cysteine (Cys), glutathione (GSH), and phytochelatins (PCs) in rice tissues at the booting and filling stages.

		Booting Stage			Filling Stage		
		Cys	GSH	PCs	Cys	GSH	PCs
Grain	CK	N	N	N	67.6 ± 11.2 a	75.4 ± 10.0 b	79.26 ± 5.4 b
	S	N	N	N	66.0 ± 9.5 a	79.2 ± 17.2 b	92.42 ± 4.3 a
	W	N	N	N	66.3 ± 6.7 a	95.3 ± 8.3 a	81.11 ± 8.7 ab
	WS	N	N	N	62.3 ± 8.2 a	68.3 ± 7.5 b	75.48 ± 6.5 b
Leaf	CK	Nd	Nd	61.3 ± 9.7 a	Nd	Nd	81.74 ± 18.2 b
	S	Nd	Nd	76.2 ± 8.4 a	Nd	Nd	165.1 ± 31.5 a
	W	Nd	Nd	61.3 ± 12.5 a	Nd	Nd	79.7 ± 8.9 b
	WS	Nd	Nd	63.2 ± 3.9 a	Nd	Nd	84.5 ± 7.3 b
Stem	CK	Nd	Nd	52.1 ± 9.2 a	Nd	Nd	81.7 ± 6.5 a
	S	Nd	Nd	58.6 ± 6.3 a	Nd	Nd	86.4 ± 17.1 a
	W	Nd	Nd	59.5 ± 11.5 a	Nd	Nd	77.5 ± 5.0 a
	WS	Nd	Nd	61.2 ± 7.7 a	Nd	Nd	82.8 ± 10.6 a
Root	CK	70.1 ± 16.2 a	Nd	51.1 ± 5.4 a	65.6 ± 5.1 b	67.1 ± 6.4 b	138.9 ± 16.2 a
	S	67.2 ± 11.1 a	Nd	54.2 ± 9.3 a	78.1 ± 7.2 a	76.5 ± 3.3 a	147.4 ± 21.5 a
	W	76.4 ± 9.6 a	Nd	59.2 ± 5.7 a	62.9 ± 5.9 b	65.1 ± 5.2 b	135.0 ± 22.4 a
	WS	75.4 ± 7.4 a	Nd	57.6 ± 11.3 a	64.6 ± 13.3 ab	63.4 ± 4.1 b	136.9 ± 12.7 a

CK, without addition; W, addition of wheat straw; S, addition of sulfate; WS, combined application of wheat straw and sulfate; N, no grain grew at the booting stage; Nd, value below the detection limit; the concentration of PCs corresponds to the cumulative sum of PC_2_, PC_3_, and PC_4_; different letters represent significant differences between treatments at *p* < 0.05.

## Data Availability

The original contributions presented in this study are included in the article. Further inquiries can be directed to the corresponding author(s).
